# Supplementary material for: Single-cell sequencing reveals clonal expansions of pro-inflammatory synovial CD8 T cells expressing tissue-homing receptors in psoriatic arthritis
Source: Nat Commun. 2020 Sep 21;11:4767. doi: 10.1038/s41467-020-18513-6 (PMC7505844; doi:10.1038/s41467-020-18513-6)
Supplement: Supplementary file 1 — Supplementary Information [file 41467_2020_18513_MOESM1_ESM.pdf]

Single-cell sequencing reveals clonal expansions of pro-inflammatory synovial CD8 T cells expressing tissue-homing receptors in psoriatic arthritis

Penkava et al.

Supplementary Figure 1: CyTOF Pre-processing and annotation

a

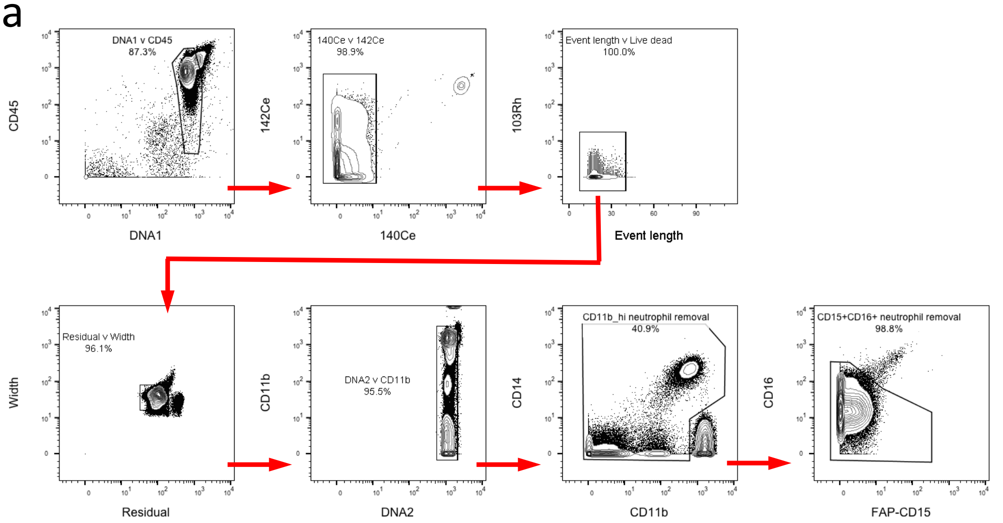

b

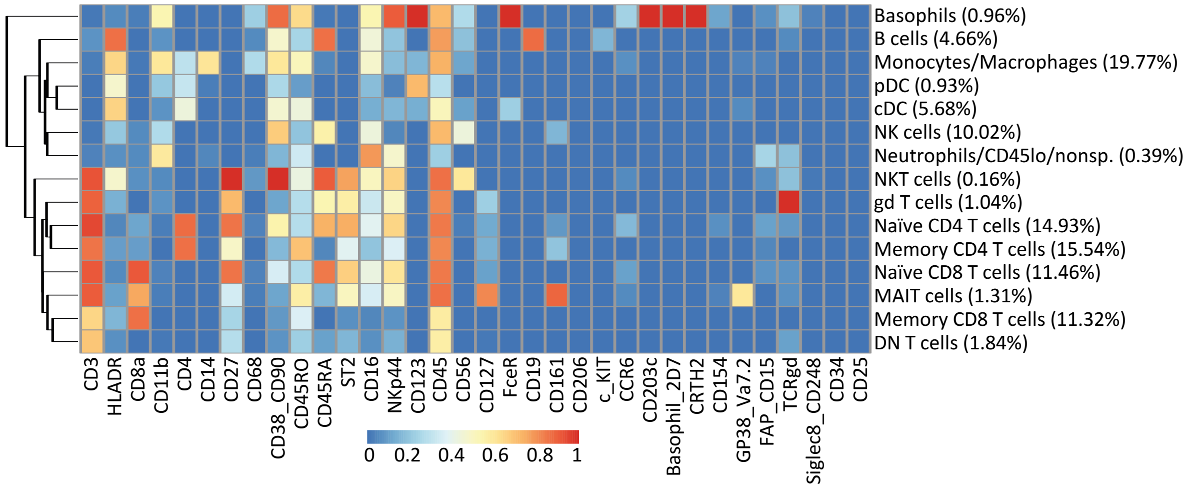

c

Cluster name	Heat map defining markers
Naive CD8 T cells	CD45+ CD3+ CD56- CD8a+ CD4- CD45RA+ CD27+
Naive CD4 T cells	CD45+ CD3+ CD56- CD4+ CD8a- CD45RA+ CD27+
Memory CD8 T cells	CD45+ CD3+ CD56- CD8a+ CD4- CD45RO+ (or CD45RA+ CD27-)
Memory CD4 T cells	CD45+ CD3+ CD56- CD4+ CD8a- CD45RO+ (or CD45RA+ CD27-)
Monocytes / Macrophages	CD45+ CD11b+ CD68+ HLA-DR+ CD4lo CD14+ (or CD16+)
B cells	CD45+ CD19+ HLA-DR+ CD45RA+
Basophils	CD45+ CD123+ FceR+ CRHT2+ CD203+ 2D7+ CD11b+ HLA-DR-
DN T cells	CD45+ CD3+ CD56- CD8a- CD4-
gd T cells	CD45+ CD3+ TCRgd+ CD8a- CD4-
MAIT cells	CD45+ CD3+ CD56- CD8a+ CD161hi Va7.2+
NK cells	CD45+ CD3- CD56+ CD161+ CD16lo/+ HLA-DR- CD45RA+
NKT cells	CD45+ CD3+ CD56+
pDC	CD45+ CD123+ HLA-DR+ CD14- CD11b- CD4lo
cDC	CD45+ CD123- HLA-DR+ CD14- CD11b- CD4lo FceR+

Supplementary Figure 1: CyTOF Pre-processing and annotation. a. CyTOF data were pre-processed to remove debris, normalisation beads, non-specific staining and neutrophils. Biaxial manual gating was performed using FlowJo software (representative sample shown). **b.** Representative heatmap from one PsA patient, showing the median arcsinh-transformed marker intensity normalised to a 0 to 1 range, limited to the markers used by the FlowSOM algorithm for clustering of CyTOF data. The dendrogram indicates the hierarchical similarity of the cell clusters (Euclidean distance, average linkage). The percentage indicates the size of each of the cell clusters for the combined matched blood and synovial fluid samples. **c.** Defining CyTOF markers from the heatmap in (b) that led to annotation and merging.

Supplementary Figure 2: Peripheral blood and synovial fluid mononuclear cell populations identified by 10x 3-prime single cell sequencing.

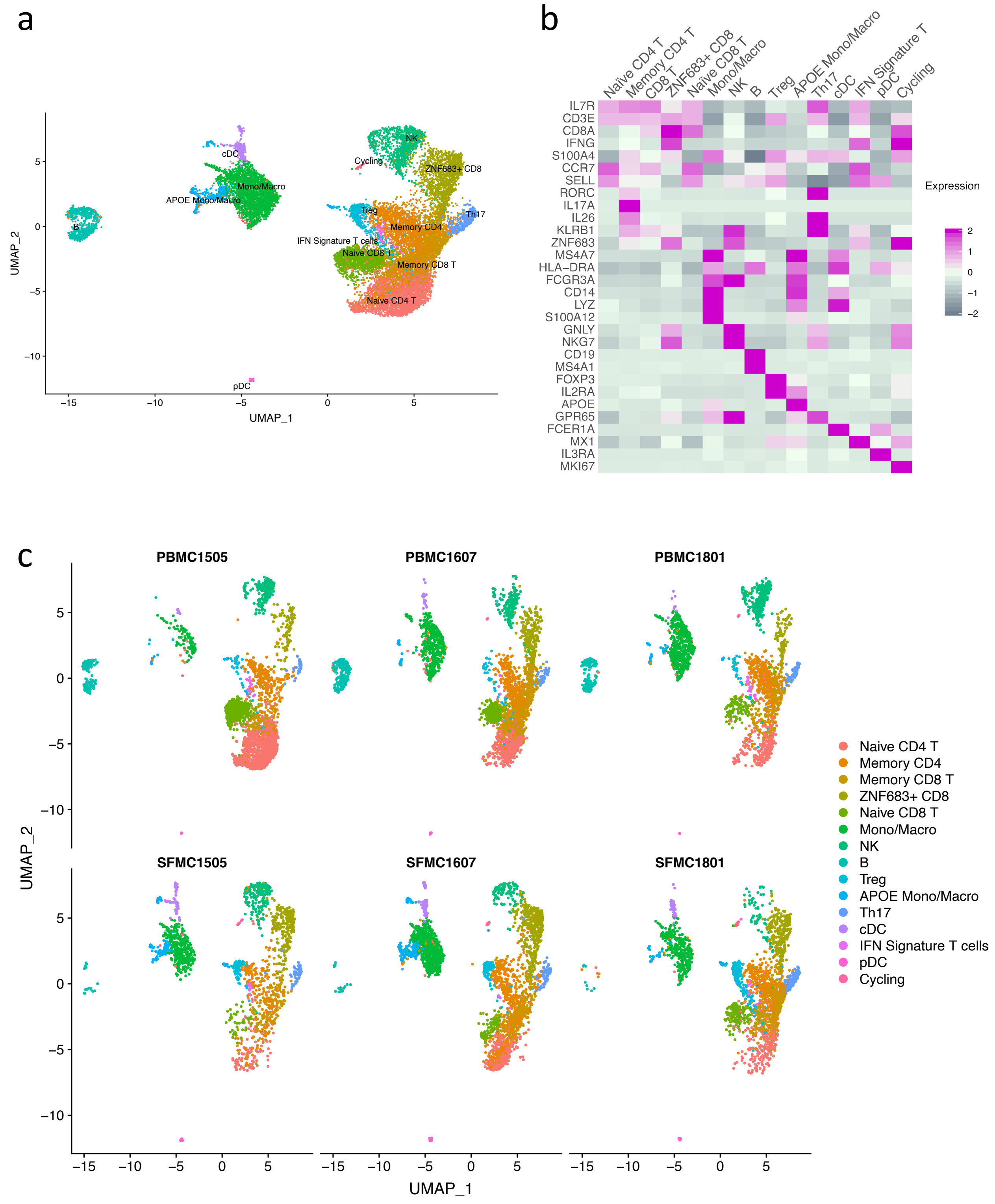

Supplementary Figure 2: Peripheral blood and synovial fluid mononuclear cell populations identified by 10x 3-prime single cell sequencing. a. UMAP of integrated PsA paired PBMC and SFMC data from three donors generated by 10x 3' sequencing. **b.** Heatmap of gene expression for clusters in (a). The relative expression of marker genes (rows) across cell clusters (columns) is shown. **c.** UMAP from (a) split by donor and sample type.

Supplementary Figure 3: Gating strategies used for cell sorting

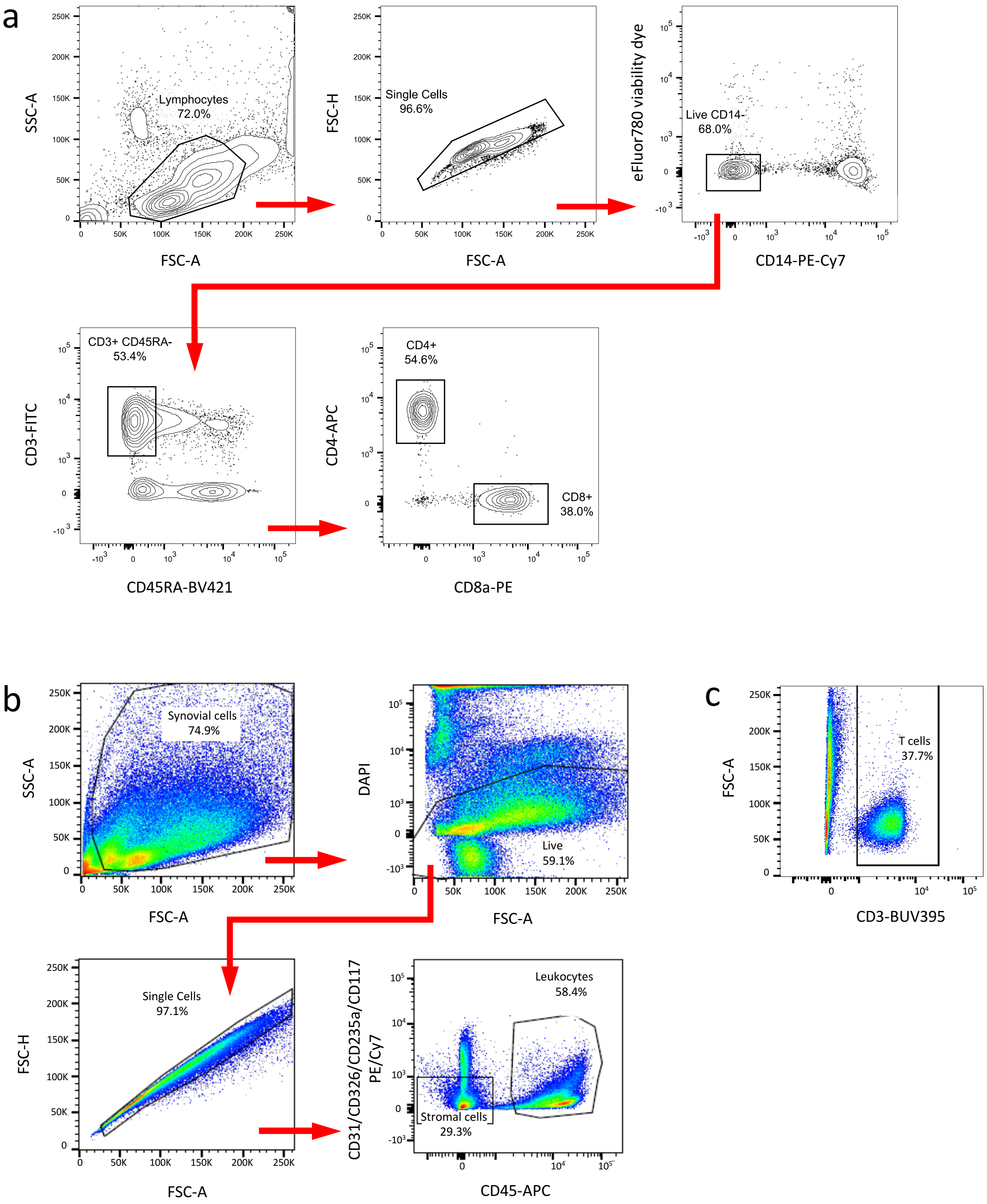

Supplementary Figure 3: Gating strategies used for cell sorting. **a.** Gating strategy used to sort live CD14-CD3+CD45RA-CD8-CD4+ and CD14-CD3+CD45RA-CD4-CD8+ memory T cells from peripheral blood and synovial fluid mononuclear cells prior to single cell 10x 5 prime and Smart-seq2 RNA sequencing. Post-sort population purity was > 99%. Presented on Figures 2a, 2b, 2c, 2d, 2f, 2g, 2h, 3, 4 and 5. **b.** Gating strategy used to sort live CD45+ leukocytes from synovial tissue cells prior to single cell 10x 5 prime RNA sequencing. Presented on Figures 2a, 2b and 2e after further subsetting based on RNA expression described in Supplementary Figure 4a. **c.** Post-sorting percentage of CD3+ T cells from leukocyte gate in (b). Plots in (a), (b) and (c) are from representative samples.

Supplementary Figure 4: Leucocyte cell clusters identified from synovial tissue biopsies of 2 PSA patients using 5’ 10x single cell RNA sequencing.

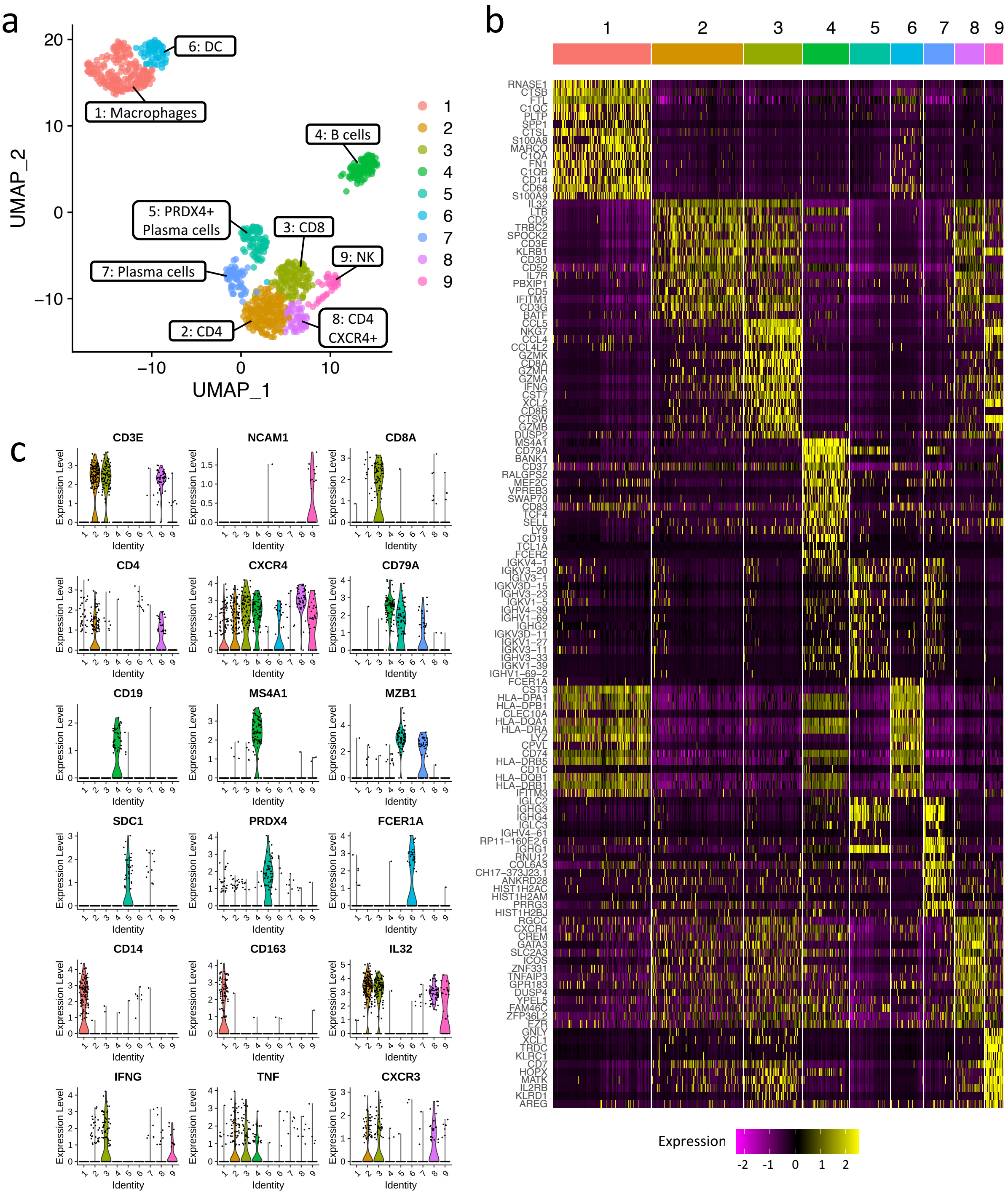

Supplementary Figure 4: Leucocyte cell clusters identified from synovial tissue biopsies of 2 PSA patients using 5’ 10x single cell RNA sequencing. CD45+ sorted cells from 2 PSA knee synovial tissue biopsies were quality control filtered, normalised using SCTransform, merged into a single 10x 5 prime gene expression matrix and clustered based on the top 2000 variable genes from the SCT assay (see methods) to identify 9 cell clusters. **a.** UMAP representation of clusters identified in the merged dataset of both PSA patients. Each dot is a cell and clusters are numbered from largest to smallest. Cells expressing CD3E from clusters 2, 3 and 8 were used in downstream T cell analysis. **b.** Heatmap of the top 15 differentially overexpressed genes per cluster calculated using a 2-sided Wilcoxon Rank Sum test and having an adjusted p-value < 0.05. P-value adjustment performed using Bonferroni correction based on total number of genes in the dataset. Genes are sorted from highest to lowest average logFC within each cluster and visualized using scaled data from the log normalised RNA assay. **c.** Violin plots of key cluster markers based on log normalized RNA.

Supplementary Figure 5: Extended heatmap of genes associated with the 16 clusters identified from the unified scRNAseq analysis of CD4 and CD8 T cells from all sample types (peripheral blood, synovial fluid and synovial tissue).

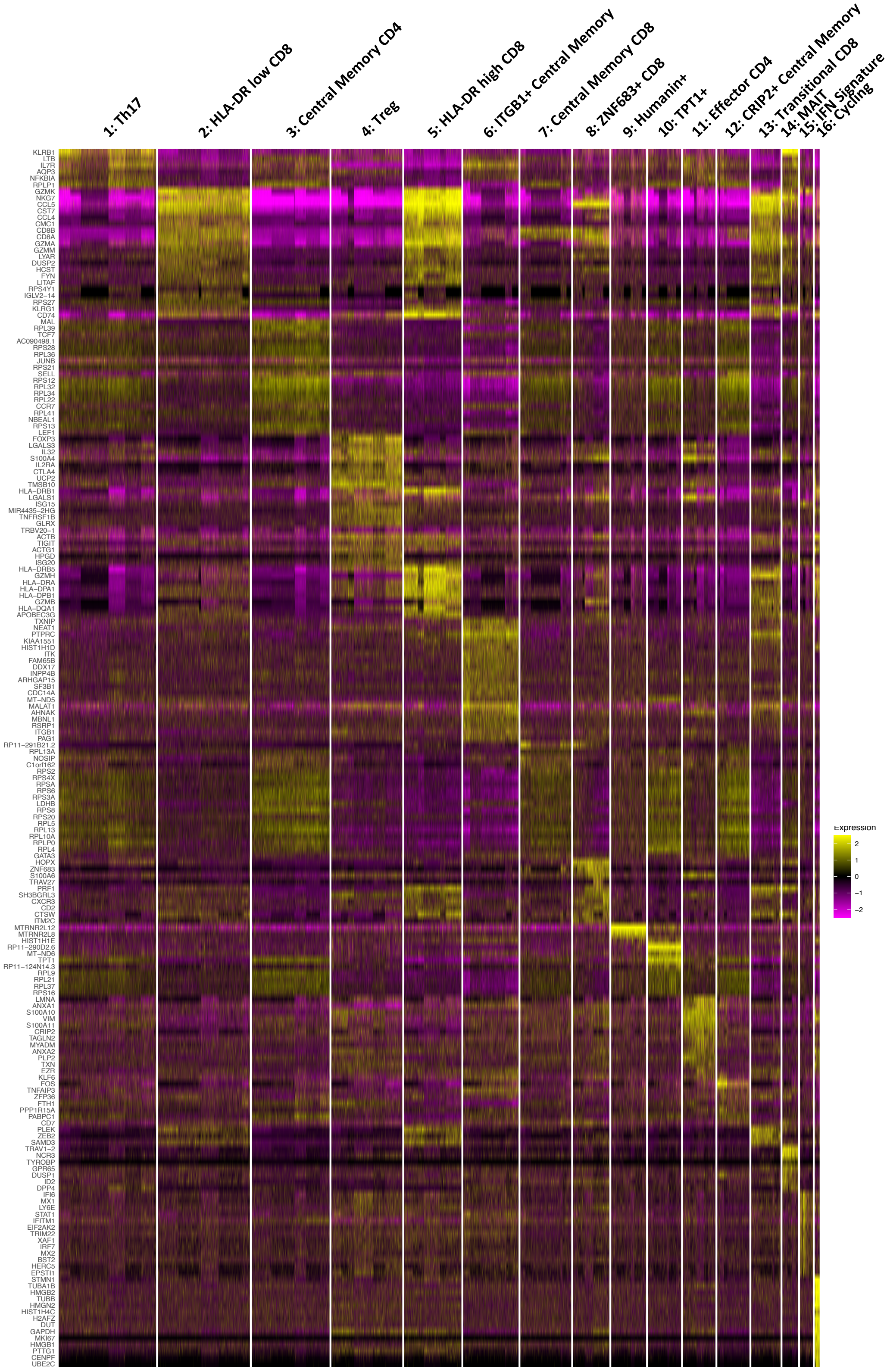

Supplementary Figure 5: Extended heatmap of genes associated with the 16 clusters identified from integrated scRNAseq analysis of CD4 and CD8 T cells from all sample types (peripheral blood, synovial fluid and synovial tissue). The top 15 differentially overexpressed genes per cluster calculated using a 2-sided Wilcoxon Rank Sum test and having an adjusted *p*-value (calculated using Bonferroni correction based on total number of genes) < 0.05 are shown. Genes are sorted by highest to lowest average logFC of normalized RNA expression within each cluster and visualized using scaled data from the SCT assay. Integrated data from 5 PsA patients (3 paired blood / synovial fluid, 2 synovial tissue).

Supplementary Figure 6: Validation of merged 5'10x paired synovial fluid and peripheral blood T cell samples from 3 PsA patients.

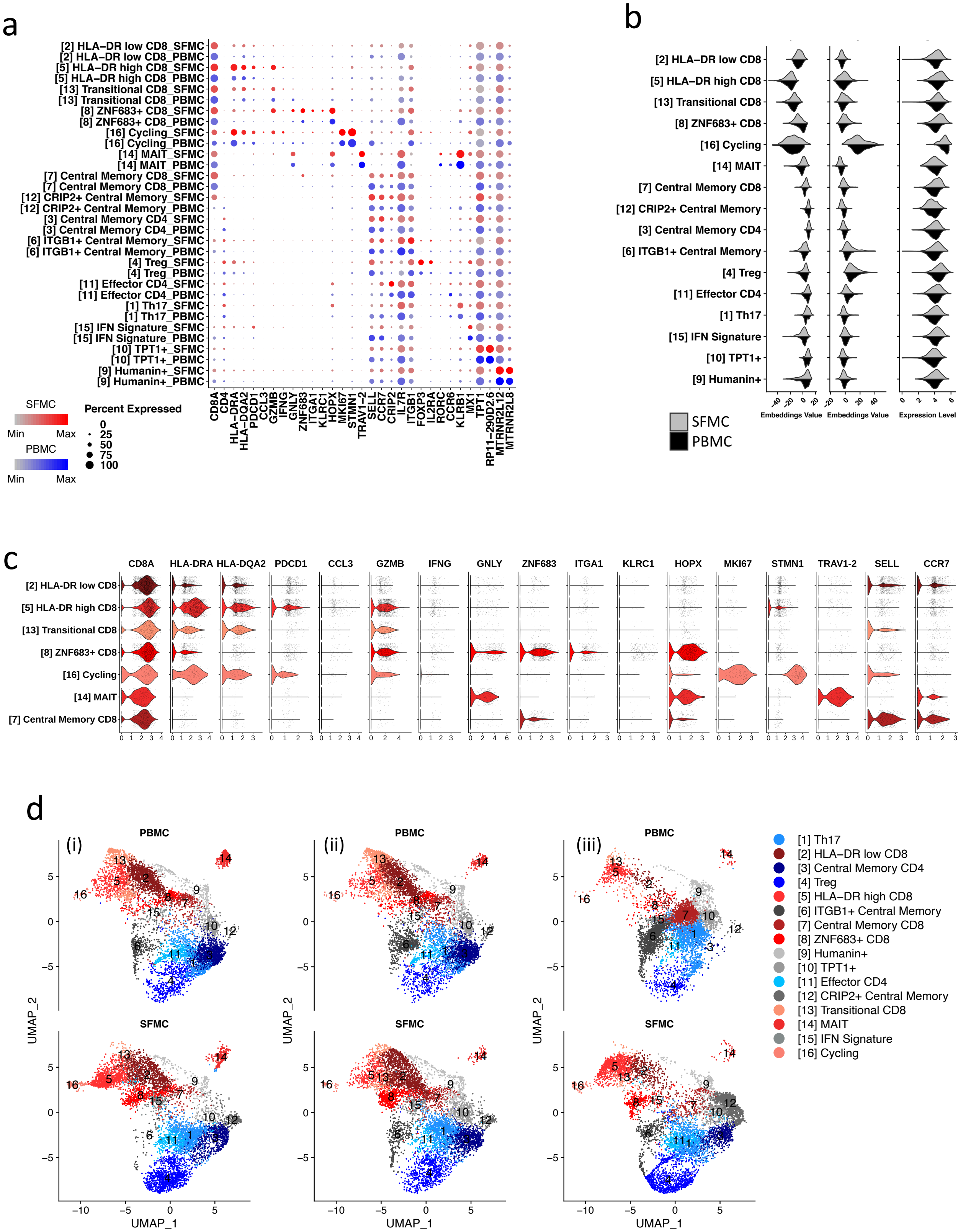

Supplementary Figure 6: Validation of merged 5'10x paired synovial fluid and peripheral blood T cell samples from 3 PsA patients. a. Key marker genes used for cluster classification compared by sample type. **b.** First 2 principal components and beta actin gene expression for each cluster by sample type. **c.** Violin plots of synovial CD8 cluster gene expression. **d.** UMAP plots showing cluster distribution of 10x dataset split by patient (i)PSA1505, (ii)PSA1607, (iii)PSA1801 and sample type.

Supplementary Figure 7: Phenotypes of individual CD8 T cell clones enriched in peripheral blood and synovial fluid.

a Peripheral blood enriched clones per patient sorted by percentage of cells with HLA-DR low phenotype

Clone	[2] HLA-DR low	[5] HLA-DR high	[13] Transitional CD8	[7] Central Memory CD8	[8] ZNF683+ CD8	[16] Cycling
1505-C4-PB	91.18%	8.82%	0.00%	0.00%	0.00%	0.00%
1505-C9-PB	85.71%	7.14%	7.14%	0.00%	0.00%	0.00%
1505-C13-PB	80.77%	7.69%	11.54%	0.00%	0.00%	0.00%
1505-C1-PB	78.69%	9.02%	10.66%	0.00%	0.00%	1.64%
1505-C8-PB	78.95%	5.26%	15.79%	0.00%	0.00%	0.00%
1505-C7-PB	66.67%	16.16%	16.16%	0.00%	0.00%	1.01%
1505-C18-PB	62.50%	18.75%	12.50%	6.25%	0.00%	0.00%
1505-C3-PB	59.26%	23.46%	17.28%	0.00%	0.00%	0.00%
1505-C16-PB	57.14%	32.14%	10.71%	0.00%	0.00%	0.00%
1505-C11-PB	55.56%	33.33%	11.11%	0.00%	0.00%	0.00%
1505-C12-PB	43.48%	21.74%	30.43%	4.35%	0.00%	0.00%
1505-C5-PB	39.62%	16.98%	43.40%	0.00%	0.00%	0.00%
1505-C14-PB	29.17%	27.08%	41.67%	0.00%	0.00%	2.08%
1505-C6-PB	15.15%	21.21%	60.61%	0.00%	0.00%	3.03%
1505-C10-PB	15.38%	7.69%	76.92%	0.00%	0.00%	0.00%
1505-C17-PB	9.09%	0.00%	90.91%	0.00%	0.00%	0.00%
1505-C15-PB	0.00%	0.00%	0.00%	100.00%	0.00%	0.00%
1505-C2-PB	0.00%	0.00%	0.00%	2.56%	97.44%	0.00%
1607-C6-PB	75.00%	0.00%	25.00%	0.00%	0.00%	0.00%
1607-C3-PB	46.67%	13.33%	40.00%	0.00%	0.00%	0.00%
1607-C4-PB	45.83%	0.00%	54.17%	0.00%	0.00%	0.00%
1607-C9-PB	44.44%	11.11%	44.44%	0.00%	0.00%	0.00%
1607-C5-PB	31.94%	11.11%	55.56%	0.00%	0.00%	1.39%
1607-C2-PB	18.18%	9.09%	72.73%	0.00%	0.00%	0.00%
1607-C8-PB	0.00%	37.50%	62.50%	0.00%	0.00%	0.00%
1607-C7-PB	0.00%	0.00%	0.00%	50.00%	50.00%	0.00%
1607-C1-PB	0.00%	4.17%	0.00%	4.17%	91.67%	0.00%

b Synovial fluid enriched clones per patient sorted by percentage of cells with HLA-DR high phenotype

Clone	[2] HLA-DR low	[5] HLA-DR high	[13] Transitional CD8	[7] Central Memory CD8	[8] ZNF683+ CD8	[16] Cycling
1505-C1-SF	10.94%	85.16%	0.00%	0.00%	0.00%	3.91%
1505-C9-SF	17.39%	82.61%	0.00%	0.00%	0.00%	0.00%
1505-C8-SF	5.88%	82.35%	0.00%	0.00%	0.00%	11.76%
1505-C19-SF	17.65%	82.35%	0.00%	0.00%	0.00%	0.00%
1505-C20-SF	13.64%	81.82%	4.55%	0.00%	0.00%	0.00%
1505-C3-SF	11.54%	80.77%	1.28%	0.00%	0.00%	6.41%
1505-C11-SF	10.00%	80.00%	0.00%	0.00%	0.00%	10.00%
1505-C6-SF	16.22%	78.38%	0.00%	0.00%	0.00%	5.41%
1505-C14-SF	5.56%	77.78%	0.00%	0.00%	0.00%	16.67%
1505-C12-SF	11.54%	76.92%	0.00%	0.00%	0.00%	11.54%
1505-C16-SF	7.69%	76.92%	0.00%	0.00%	0.00%	15.38%
1505-C4-SF	17.86%	76.79%	0.00%	0.00%	0.00%	5.36%
1505-C2-SF	20.83%	76.04%	0.00%	0.00%	1.04%	2.08%
1505-C15-SF	16.67%	75.00%	0.00%	0.00%	0.00%	8.33%
1505-C13-SF	13.33%	73.33%	6.67%	0.00%	0.00%	6.67%
1505-C5-SF	24.59%	68.85%	0.00%	0.00%	0.00%	6.56%
1505-C7-SF	26.09%	60.87%	4.35%	0.00%	0.00%	8.70%
1505-C18-SF	33.33%	58.33%	0.00%	0.00%	0.00%	8.33%
1505-C10-SF	12.50%	0.00%	0.00%	0.00%	87.50%	0.00%
1607-C4-SF	10.00%	70.00%	10.00%	0.00%	10.00%	0.00%
1607-C2-SF	33.33%	48.81%	7.14%	1.19%	3.57%	5.95%
1607-C6-SF	68.97%	13.79%	10.34%	3.45%	3.45%	0.00%
1607-C8-SF	48.00%	9.33%	22.67%	18.67%	1.33%	0.00%
1607-C10-SF	34.38%	6.25%	3.13%	56.25%	0.00%	0.00%
1607-C1-SF	4.28%	0.39%	5.84%	0.39%	88.33%	0.78%
1607-C7-SF	100.00%	0.00%	0.00%	0.00%	0.00%	0.00%
1607-C3-SF	0.00%	0.00%	0.00%	0.00%	98.11%	1.89%
1607-C5-SF	0.00%	0.00%	0.00%	0.00%	100.00%	0.00%
1801-C7-SF	0.00%	100.00%	0.00%	0.00%	0.00%	0.00%
1801-C2-SF	0.00%	94.94%	2.53%	0.00%	0.00%	2.53%
1801-C5-SF	6.82%	86.36%	4.55%	0.00%	0.00%	2.27%
1801-C1-SF	6.35%	84.13%	7.14%	0.00%	0.00%	2.38%
1801-C4-SF	39.39%	54.55%	3.03%	0.00%	0.00%	3.03%
1801-C3-SF	3.57%	53.57%	17.86%	0.00%	21.43%	3.57%
1801-C6-SF	5.88%	0.00%	5.88%	44.12%	41.18%	2.94%

Percentage of cluster

0% 100%

Supplementary figure 7: Phenotypes of individual CD8 T cell clones enriched in peripheral blood and synovial fluid. Heatmaps depicting for each CD8 clone enriched in either **a.** peripheral blood or **b.** synovial fluid, the proportions of that clone belonging to different CD8 T cell clusters. Clones are sorted for each patient by the percentage of cells belonging to the HLA-DR low cluster in peripheral blood, and HLA-DR high cluster in synovial fluid. Where more than 50% of a clone is associated with a specific cluster, text is white. Cell counts per clone per cluster are derived from the peripheral blood and synovial fluid 10x 5 prime datasets from 3 psoriatic arthritis patients: PSA1505, PSA1607 and PSA1801.

Supplementary Figure 8: PSA1607 10x and SS2 data integration

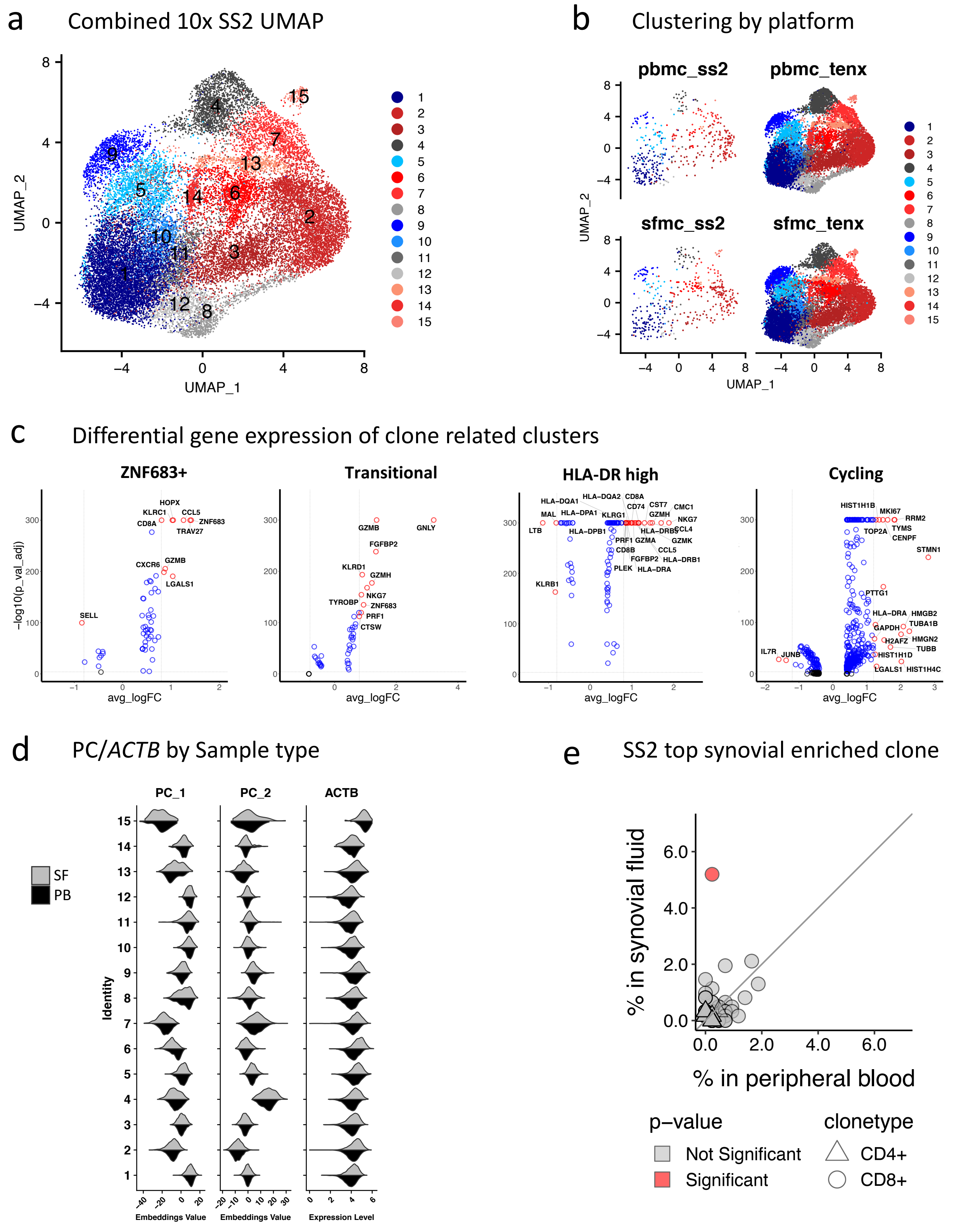

Supplementary Figure 8: PSA1607 10x and SS2 data integration. a. UMAP clustering after integration of PSA1607 SFMC and PBMC derived T cells from 10x and SS2 datasets. **b.** Post integration UMAP distribution of cells by platform and sample type. **c.** Volcano plots of clusters in (a) associated with most enriched clone. Statistics calculated using 2-sided Wilcoxon Rank Sum test with Bonferroni correction, labelling cut-offs for average logFC (±1.2 Cycling, ±0.8 remaining clusters) and adjusted *p*-value (<= 1E-04) indicated by dotted lines. **d.** First 2 principal components and *ACTB* expression compared by sample type post integration. **e.** Clones enriched (2-sided Fisher's exact test, adjusted *p*-value <= 0.05, Benjamini-Hochberg correction) within synovial fluid and peripheral blood for patient PSA1607 based on SS2 sequencing (compare with Figure 3e). Source data are provided as a Source Data file.

Supplementary Figure 9: Synovial CRG-1 expresses tissue homing and pro-inflammatory gene programs.

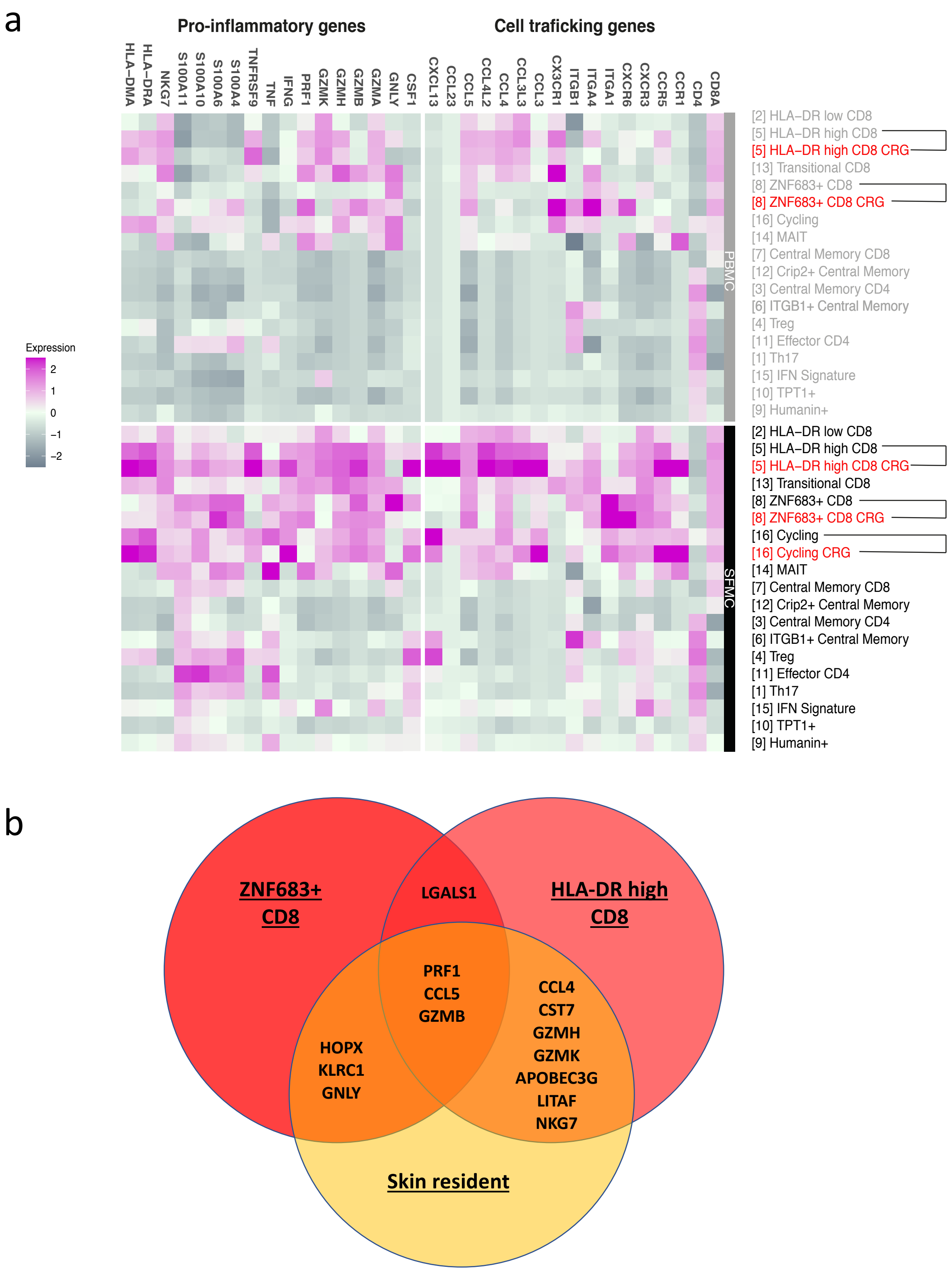

Supplementary Figure 9: Synovial CRG-1 expresses tissue homing and pro-inflammatory gene programs.
a. Heatmap of pro-inflammatory and homing marker gene expression in PBMC and SFMC derived CD4 and CD8 T cells from 5' 10x integrated dataset of 3 PsA patients. Compares CRG-1 cells subsetted from clusters 5, 8 and 16 with remaining cells from those clusters. No CD8 cycling cells belonging to CRG-1 originated from PBMC. **b.** Overlap of upregulated genes in ZFN683+ CD8 and HLA-DR high CD8 clusters (2-sided Wilcoxon rank sum test with Bonferroni correction, adjusted *p*-value <= 0.05) from this paper with previously described genes upregulated in tissue-resident epidermal CD49a+ CD8 T skin cells "poised for cytotoxic function", Cheuk, S. et al. (2017) [12]

Supplementary Table 1: PsA patient demographics

Matched peripheral blood and synovial fluid samples for CyTOF

Patient	Age at study entry	Gender	Medications
PsA1505	35	M	nil
PsA1522	56	M	nil
PsA1718	31	F	nil
PsA1719	34	M	nil
PsA1724	65	M	Leflunomide, Etanercept
PsA1725	49	M	Methotrexate
PsA1728	72	F	nil
PsA1733	61	M	nil
PsA1801	53	F	nil
PsA1607	42	M	nil

Matched peripheral blood and synovial fluid samples for droplet based single-cell RNA Sequencing

Patient	Age at study entry	Gender	Medications
PsA1801	54	F	nil
PsA1505	35	M	nil
PsA1607	42	M	nil

Synovial tissue biopsy samples for droplet based single-cell RNA Sequencing

Patient	Age at study entry	Gender	Medications
N_PsA4040	40	M	nil
N_PsA245	59	M	nil

Matched peripheral blood and synovial fluid samples for plate based single-cell RNA Sequencing

Patient	Age at study entry	Gender	Medications
PsA1607	42	M	nil
PsA1718	31	F	nil
PsA1719	33	M	nil
PsA1728	72	F	nil

Matched plasma and synovial fluid supernatant for protein quantification

Patient	Age at study entry	Gender	Medications
PsA1512	26	F	nil
PsA1718	31	F	nil
PsA1719	33	M	nil
PsA1724	65	M	Leflunomide, Etanercept
PsA1728	72	F	nil
PsA1505	35	M	nil
PsA1607	42	M	nil
PsA1725	49	M	Methotrexate
PsA1733	61	M	nil
PsA1801	53	F	nil
PsA1802	33	F	Methotrexate

Supplementary Table 2: CyTOF antibodies

Target	Metal	Clone	Supplier	catalogue number	Dilution
Basophil_2D7	158Gd	2D7	Thermo Fisher (eBioscience)	Custom order of 12-9748-42	0.2mg/ml
CCR6	155Gd	11A9	BD Biosciences	559560	0.2mg/ml
CD117 (c-KIT)	176Yb	104D2	BioLegend	313201	0.2mg/ml
CD11b	209bi	ICRF44	Fluidigm	3209003B	0.2mg/ml
CD123	151Eu	6H6	Thermo Fisher (eBioscience)	14-1239-82	0.2mg/ml
CD127	163Dy	A019D5	BioLegend	351302	0.2mg/ml
CD14	160Gd	61D3	Thermo Fisher (eBioscience)	14-0149-82	0.2mg/ml
CD15	144Nd	W6D3	BioLegend	323002	0.2mg/ml
CD154	150Nd	24-31	BioLegend	310835	0.2mg/ml
CD16	148Nd	3G8	Thermo Fisher (eBioscience)	16-0166-85	0.2mg/ml
CD161	161Dy	DX12	BD Biosciences	556079	0.2mg/ml
CD19	142Nd	HIB19	Thermo Fisher (eBioscience)	14-0199-82	0.2mg/ml
CD203c	156Gd	NP4D6	Thermo Fisher (eBioscience)	MA1-10138	0.2mg/ml
CD206	154Sm	15-2	BioLegend	321101	0.2mg/ml
CD248	141Pr	B1/35	BD Biosciences	564993	0.2mg/ml
CD25	149Sm	BC96	Thermo Fisher (eBioscience)	14-0259-82	0.2mg/ml
CD27	167Er	O323	Thermo Fisher (eBioscience)	14-0279-82	0.2mg/ml
CD3	170Er	UCHT1	Thermo Fisher (eBioscience)	14-0038-82	0.2mg/ml
CD34	147Sm	4H11	Thermo Fisher (eBioscience)	14-0349-82	0.2mg/ml
CD38	172Yb	HIT2	Thermo Fisher (eBioscience)	14-0389-82	0.2mg/ml
CD4	145Nd	RPA-T4	Thermo Fisher (eBioscience)	14-0049-82	0.2mg/ml
CD45	89Y	HI30	Fluidigm	3089003B	0.2mg/ml
CD45RA	169Tm	HI100	Thermo Fisher (eBioscience)	14-0458-82	0.2mg/ml
CD45RO	171Yb	UCHL1	BioLegend	304202	0.2mg/ml
CD56	173Yb	HCD56	BioLegend	318302	0.2mg/ml
CD68	159Tb	FA-11	BioLegend	137001	0.2mg/ml
CD8a	146Nd	RPA-T8	Thermo Fisher (eBioscience)	Custom order of 17-0088-42	0.2mg/ml
CD90	172Yb	5E10	BioLegend	328101	0.2mg/ml
CRTH2	152Sm	BM16	BD Biosciences	558412	0.2mg/ml
FAP	144Nd	F11-24	Thermo Fisher (eBioscience)	BMS168	0.2mg/ml
FceR	165Ho	AER-37 (CRA-1)	BioLegend	334602	0.2mg/ml
gp38	143Nd	NC-08	BioLegend	337002	0.2mg/ml
HLA-DR	174Yb	LN3	Thermo Fisher (eBioscience)	14-9956-82	0.2mg/ml
NKp44	166Er	P44-8	BioLegend	325101	0.2mg/ml
Siglec-8	141Pr	7C9	BioLegend	347102	0.2mg/ml
ST2	168Er	polyclonal	R&D Systems	AF523	0.2mg/ml
TCRgd	164Dy	B1	BioLegend	331202	0.2mg/ml
Va7.2	143Nd	3C10	BioLegend	351702	0.2mg/ml
