# Supplementary material for: Single-cell sequencing reveals clonal expansions of pro-inflammatory synovial CD8 T cells expressing tissue-homing receptors in psoriatic arthritis
Source: Nat Commun. 2020 Sep 21;11:4767. doi: 10.1038/s41467-020-18513-6 (PMC7505844; doi:10.1038/s41467-020-18513-6)
Supplement: Supplementary file 3 — Reporting Summary [file 41467_2020_18513_MOESM3_ESM.pdf]

nature research

Corresponding author(s): Hussein Al-Mossawi
Sam Behjati

Last updated by author(s): Aug 19, 2020

Reporting Summary

Nature Research wishes to improve the reproducibility of the work that we publish. This form provides structure for consistency and transparency in reporting. For further information on Nature Research policies, see our Editorial Policies and the Editorial Policy Checklist.

Statistics

For all statistical analyses, confirm that the following items are present in the figure legend, table legend, main text, or Methods section.

n/a	Confirmed	
☐	☒	The exact sample size (*n*) for each experimental group/condition, given as a discrete number and unit of measurement
☐	☒	A statement on whether measurements were taken from distinct samples or whether the same sample was measured repeatedly
☐	☒	The statistical test(s) used AND whether they are one- or two-sided<br>*Only common tests should be described solely by name; describe more complex techniques in the Methods section.*
☒	☐	A description of all covariates tested
☐	☒	A description of any assumptions or corrections, such as tests of normality and adjustment for multiple comparisons
☒	☐	A full description of the statistical parameters including central tendency (e.g. means) or other basic estimates (e.g. regression coefficient) AND variation (e.g. standard deviation) or associated estimates of uncertainty (e.g. confidence intervals)
☐	☒	For null hypothesis testing, the test statistic (e.g. *F*, *t*, *r*) with confidence intervals, effect sizes, degrees of freedom and *P* value noted<br>*Give P values as exact values whenever suitable.*
☒	☐	For Bayesian analysis, information on the choice of priors and Markov chain Monte Carlo settings
☒	☐	For hierarchical and complex designs, identification of the appropriate level for tests and full reporting of outcomes
☒	☐	Estimates of effect sizes (e.g. Cohen's *d*, Pearson's *r*), indicating how they were calculated

Our web collection on statistics for biologists contains articles on many of the points above.

Software and code

Policy information about availability of computer code

Data collection	BD FACSDiva (version 8.0.1; https://www.bdbiosciences.com)
Data analysis	Cellranger (versions 2.1, 3.0.1, 3.0.2; https://www.10xgenomics.com) was used for droplet based gene expression and TCR mapping. For plate-based Smart-seq 2 gene expression was mapped with Kallisto (version 0.43.1; https://pachterlab.github.io/kallisto) and summarised using Scater (version 1.6.3; https://doi.org/doi:10.18129/B9.bioc.scater) while TCR sequences were constructed using TraCeR (https://github.com/teichlab/tracer) and mapped to V,D, J genes and CDR3 using the online IMGT-HighVQuest (version 3.4.13; http://www.imgt.org/HighV-QUEST) tool.<br><br>Single cell analysis was performed using R (version 3.6.1; https://www.r-project.org) with default Mersenne-Twister and L'Ecuyer-CMRG random number generators for single and parallel operations respectively. Seurat recommended pipelines were followed using Seurat (versions 3.0.0, 3.1.4; https://satijalab.org/seurat) and sctransform (version 0.2.1; https://github.com/ChristophH/sctransform) packages, and UMAP plots generated using umap-learn (0.3.10; https://github.com/lmcinnes/umap) executed within a Python (version 3.6.10; https://www.python.org) numpy (version 1.18.1; https://numpy.org) environment. The jsonlite (version 1.6.1; https://arxiv.org/abs/1403.2805) R package was used to read in TCR information generated by Cellranger, with additional TCR analysis performed by GLIPH (version 1.0; https://github.com/immunoengineer/gliph) and VDJtools (version 1.2.1; http://journals.plos.org/ploscompbiol/article?id=10.1371/journal.pcbi.1004503).<br><br>FlowJo (version 10.3; https://www.flowjo.com), LegendPlex (version 8.0; http://www.vigenetech.com/LEGENDplex7.htm), and FlowSOM version 1.14.0 (https://doi.org/10.1002/cyto.a.22625) were used for flow cytometry, protein quantification and CyTOF analysis respectively.

For manuscripts utilizing custom algorithms or software that are central to the research but not yet described in published literature, software must be made available to editors and reviewers. We strongly encourage code deposition in a community repository (e.g. GitHub). See the Nature Research guidelines for submitting code & software for further information.

Data

Policy information about availability of data

All manuscripts must include a data availability statement. This statement should provide the following information, where applicable:

- Accession codes, unique identifiers, or web links for publicly available datasets
- A list of figures that have associated raw data
- A description of any restrictions on data availability

The datasets generated during and/or analysed during the current study are available from the corresponding author on reasonable request. Sequencing data that support the findings of this study have been deposited in ArrayExpress with the accession code E-MTAB-9492 (https://www.ebi.ac.uk/arrayexpress/experiments/E-MTAB-9492) and in the European Genome-phenome Archive (EGA) with accession code EGAS00001002104 (https://ega-archive.org/studies/EGAS00001002104).

Field-specific reporting

Please select the one below that is the best fit for your research. If you are not sure, read the appropriate sections before making your selection.

☒ Life sciences ☐ Behavioural & social sciences ☐ Ecological, evolutionary & environmental sciences

For a reference copy of the document with all sections, see nature.com/documents/nr-reporting-summary-flat.pdf

Life sciences study design

All studies must disclose on these points even when the disclosure is negative.

Sample size	Sample sizes were determined with reference to previously published single cell sequencing data (https://www.nature.com/articles/s41467-017-02659-x), which were shown to be of sufficient size to discriminate between cell types.
Data exclusions	Cells with a high proportion of mitochondrial gene transcripts were deemed to be dying and excluded. Multiplets or empty droplets were also excluded according to the number of genes and gene transcripts observed. Where TCR data was available, cells with multiple beta chains (and/or more than 2 alpha chains) were deemed to be multiplets based on the principle of allelic exclusion. For peripheral blood and synovial fluid samples, cells were further excluded if they expressed both CD4 and CD8 transcripts given the sorting strategy used. After the filtration of dying cells and multiplets, where appropriate and as detailed in the manuscript, cells were further randomly subsampled to an equal number from each patient and sample type. Exclusion criteria were pre-established based on previous literature, and adjusted according to the observed variance within samples.
Replication	Single cell sequencing of T cells isolated from synovial fluid and blood was performed 3 times for 10x sequencing and 4 times for SS2 sequencing. Cell numbers sequenced and cell types identified were comparable between samples processed using each platform. The finding of expanded clones within synovial fluid was reproduced in all three 10x processed samples. Single cell sequencing of leukocytes isolated from synovial tissue was performed twice, identifying the presence of both CD4 and CD8 T cells within both tissue samples in similar proportions. CyTOF and chemokine protein measurement experiments were each performed 10 and 11 times respectivley. All replication attempts were successful and all data was included
Randomization	Given this was a prospective observational study, randomization was not necessary.
Blinding	Given this was a prospective observational study, blinding was not necessary.

Reporting for specific materials, systems and methods

We require information from authors about some types of materials, experimental systems and methods used in many studies. Here, indicate whether each material, system or method listed is relevant to your study. If you are not sure if a list item applies to your research, read the appropriate section before selecting a response.

Materials & experimental systems

n/a	Involved in the study	
☐	☒	Antibodies
☒	☐	Eukaryotic cell lines
☒	☐	Palaeontology and archaeology
☒	☐	Animals and other organisms
☐	☒	Human research participants
☒	☐	Clinical data
☒	☐	Dual use research of concern

Methods

n/a	Involved in the study	
☒	☐	ChIP-seq
☐	☒	Flow cytometry
☒	☐	MRI-based neuroimaging

Antibodies

Antibodies used

For CyTOF antibodies please refer to Supplementary Table 1.

Antibodies for sorting of synovial fluid and blood mononuclear cells prior to single cell sequencing:
CD3-FITC (SK7),1:50,344804 (BioLegend);
CD4-APC (RPA-T4),1:50,300514 (BioLegend);
CD8a-PE (RPA-T8),1:50,301008 (BioLegend);
CD45RA-BV421 (HI100),1:50,304130 (BioLegend);
eFluor780 viability dye,1:250,65-0865-18 (eBioscience).

Antibodies for sorting of synovial tissue cells prior to sequencing:
CD31-PeCy7 (WM59), 1:200, 303117 (BioLegend);
CD326- PeCy7 (9C4), 1:50 324221 (BioLegend);
CD235a- PeCy7 (HI264), 1:20, 349111 (BioLegend);
CD117- PeCy7 (104D2), 1:50, 313211 (BioLegend);
CD45-APC (2D1), 1:10, 340190 (BD Bioscience);
CD3-BUV395 (SK7) 1:20, 564001 (BD bioscience).

Validation

CD3-FITC (SK7), catalogue number: 344804 (BioLegend)
References: Björklund &, et al. 2016. Nat Immunol. 17:451-460. ; Lima J, et al. 2016. Reprod Sci. 10.1177/1933719116653680. ; Montes de Oca M, et al. 2016. Cell Rep. 17:399-412.; Chheda ZS, et al. 2018. J Exp Med. 9:19490.

CCD3-BUV395 (SK7) catalogue number: 564001 (BD bioscience).
Ernst DN, Shih CC. CD3 complex. J Biol Regul Homeost Agents. 2000; 14(3):226-229
Kan EA, Wang CY, Wang LC, Evans RL. Noncovalently bonded subunits of 22 and 28 kd are rapidly internalized by T cells reacted with anti-Leu-4 antibody. J Immunol. 1983; 131(2):536-539.

CD4-APC (RPA-T4), catalogue number: 300514 (BioLegend)
References: André M, et al. 2010. J Immunol. 185:2710.; Koelsch K, et al. 2013. PLoS One. 8:50068.; Reichwald K, et al. 2014. PLoS One. 9:105627.; Fletcher H, et al. 2016. Nat Commun. 7: 11290.; Usero L, et al. 2016. Diabetes. 65: 2356 - 2366.; Kerstein A, et al. 2016. J Autoimmun. S0896-8411(16)30186-X.; Tang-Huau TL, et al. 2018. Nat Commun. 9:2570.; Idorn M, et al. 2018. Oncoimmunology. 7:e1412029.; Wang F, et al. 2018. Oncogenesis. 7:41.; Tang M, et al. 2019. Biomed Res Int. 2019:1050285.; Park MJ, et al. 2019. J Immunol. 203:127.; Eri Yamada et al. 2018. Cell host & microbe. 23(1):110-120 .

CD8a-PE (RPA-T8), catalogue number: 301008 (BioLegend)
References: VanSaun M, et al. 2013. PLoS One. 8:73054.;Calzoni E, et al. 2019. J Allergy Clin Immunol. 143:2317.; Hebbandi Nanjundappa R, et al. 2017. Cell. 171:655.

CD45RA-BV421 (HI100), catalogue number: 304130 (BioLegend)
References: Causi E, et al. 2015. PLoS One. 10: 0136717.; Thommen D, et al. 2015. Cancer Immunol Res. 3: 1344 - 1355.; Graav G, et al. 2016. PLoS One. 11: 0148604.; Ward D, et al. 2016. Haematologica. 101: 286 - 296.; Ryan J, et al. 2016. Proc Natl Acad Sci U S A. 113: 1286 - 1295.; Sckisel G, et al. 2017. J Immunother Cancer . 10.1186/s40425-017-0235-4.
Balakrishnan A, et al. 2017. Biol Blood Marrow Transplant. 10.1016/j.bbmt.2017.07.016.; Mukhopadhyay M, et al. 2017. J Immunol. 10.4049/jimmunol.1700953.; Lichtenegger FS, et al. 2018. Front Immunol. 9:385.; Carisey AF, et al. 2018. Curr Biol. 28:489.; Celik H, et al. 2018. Cancer Cell. 34:741.; Claireaux M, et al. 2018. MBio. 9:e00317.

eFluor780 viability dye, catalogue number: 65-0865-18 (eBioscience)
References: Ulges A et al. 2016. Proc Natl Acad Sci U S A. 113(36):10145-50.; Dan JM et al. 2016. J Immunol. 197(3):983-93.; Pollizzi KN et al. 2016 Nat Immunol. 17(6):704-11.; Roan F et al. 2016. J Immunol. 196(5):2051-62.; Brodeur TY et al. 2015. J Immunol. 2015. 195(11):5251-60.; Gray EE et al. 2011. J Immunol. 186(11):6091-5.

CD45-APC (2D1), catalogue number: 340190 (BD Bioscience)
References: Borowitz MJ et al. Am J Clin Pathol. 1993;100:534-540; Borowitz MJ et al. Blood. 1997;89:3960-3966; Centers for Disease Control. MMWR. 1988;37:377-388; Jackson A. Clin Immunol Newslett. 1990;10:43-55. Krasinskas AM et al. Am J Clin Pathol. 1998;110:797- 805; Lacombe F et al. Leukemia. 1997;11:1878- 1886; Rothe G et al. Leukemia. 1996;10:877-895; Stelzer GT et al. Cytometry. 1997;30:214-230; Stewart CC et al. Cytometry. 1997;30:231-235.

CD31-PeCy7 (WM59), catalogue number: 303117 (BioLegend)
References: Zhang W, et al. 2012. J Biol Chem. 287:34157; Ardehali R, et al. 2013. Proc Natl Acad Sci U S A. 110:3405; Holdsworth-Carson S, et al. 2014. Mol Hum Reprod. 20:250; Hebel K, et al. 2014. J Immunol. 192:5160; Donnenberg V, Donnenberg A 2015. Methods. 82: 3-11; Kokai L, et al. 2016. Aesthet Surg J. 10.1093/asj/sjw197; Costa A et al. 2018. Cancer cell. 33(3):463-479; Nguyen M et al. 2018. Cell reports. 25(13):3884-3893; Pelon F, et al. 2020. Nat Commun. 0.738888889

CD326- PeCy7 (9C4), catalogue number: 324221 (BioLegend)
Tyagi N, et al. 2016. Cancer Lett. 370: 260-267; Gradiz R, et al. 2016. Sci Rep. 6:21648; Pham K, et al. 2016. Am J Pathol. 186: 1537-1546; Slamecka J, et al. 2017. Cell Cycle. 17:330; Montoro DT, et al. 2018. Nature. 560:319; Gao Y et al. 2018. Stem cell research. 28:161-164; Dijkstra KK et al. 2018. Cell. 174(6):1586-1598; Howden S et al. 2019. Stem Cell Res. 1728:53:00; Hosseini Far H

et al. 2019. Stem Cell Res. 1727:49:00; Dangaj D, et al. 2019. Cancer Cell. 35:885; Lee HJ, et al. 2017. Oncotarget. 8:113345; Bredenkamp N, et al. 2019. Stem Cell Reports. 1.29375

CD235a- PeCy7 (HI264), catalogue number: 349111 (BioLegend)
Mitchell A, et al. 2016. PLoS One. 11:e0163582; Ludwig LS et al. 2019. Cell. 176(6):1325-1339; Abud EM et al. 2017. Neuron. 94 (2):278-293

CD117- PeCy7 (104D2), catalogue number: 313211 (BioLegend)
Ogaki S, et al. 2015. Sci Rep. 5:17297.; Gall M, et al. 2015. Mol Cancer Ther. 14: 2595 - 2605; Grove K, et al. 2016. PLoS One. 11: 0145961; Serr I, et al. 2016. Nat Commun. 7:10991; Allan J, et al. 2017. Leuk Lymphoma. 10.1080/10428194.2017.1352089; Liao Y, et al. 2018. Stem Cells Transl Med. 0.659722222.

Human research participants

Policy information about studies involving human research participants

Population characteristics

Synovial fluid and blood samples were obtained from 6 patients with a current diagnosis of Psoriatic arthritis which had not been altered from any past diagnosis. 3 were male, 3 female and none were on biological therapy at the time samples were taken. Patients from which synovial tissue samples were taken were diagnosed PsA based on PsARC and not on any active treatment with DMARDs or Biologics.

For CyTOF samples, 10 participants with a current diagnosis of psoriatic arthritis were recruited. 7 were male, 3 were female. None of the patients were receiving biologic therapy, but some were on disease-modifying anti-rheumatic therapy.

For synovial tissue chemokine quantification, 11 participants with a current diagnosis of psoriatic arthritis were recruited. 6 were male, 5 were female. None of the patients were receiving biologic therapy, but some were on disease-modifying anti-rheumatic therapy.

All patient characteristics are now included in supplementary table 1.

Recruitment

Patients were recruited prospectivley from Rheumatology clinics in Oxford for blood and synovial fluid samples and Birmingham / Newcastle for synovial tissue. Recruitment may be biased to more severe spectrum of disease presenting for joint aspiration.

Ethics oversight

Oxford Research Ethics committee (Ethics reference number 06/Q1606/139) for blood and synovial fluid samples. Synovial tissue samples were obtained with approval from relevant research ethics committees (reference number 12/NE/0251 Newcastle and West Midlands Black Country Research Ethics committee 07/H1203/57 (Birmingham)

Note that full information on the approval of the study protocol must also be provided in the manuscript.

Flow Cytometry

Plots

Confirm that:

- [x] The axis labels state the marker and fluorochrome used (e.g. CD4-FITC).
- [x] The axis scales are clearly visible. Include numbers along axes only for bottom left plot of group (a 'group' is an analysis of identical markers).
- [x] All plots are contour plots with outliers or pseudocolor plots.
- [x] A numerical value for number of cells or percentage (with statistics) is provided.

Methodology

Sample preparation

Blood and synovial fluid cell preparation for CyTOF
Whole blood or synovial fluid were fixed with high-purity paraformaldehyde within 30 minutes of sample collection. Fixed blood was lysed with Permeabilization Buffer (eBioscience). Cells were stained in Maxpar staining buffer (Fluidigm).

PBMC and SFMC cell preparation for single-cell sequencing
SFMCs and PBMCs were freshly isolated within 30 minutes of sample collection by density-gradient centrifugation using Histopaque (Sigma). For 3’ 10x experiments, cells were then immediately loaded onto a 10x chromium controller. For 5’ 10x experiments, cell suspensions were additionally stained by a panel of fluorescently conjugated antibodies in staining buffer (RNAse-free PBS, 2mM EDTA) and sorted using FACS, then loaded onto a 10x chromium controller within 4 hours of sample collection. For Smart-seq2 experiments, cell suspensions were additionally stained by a panel of fluorescently conjugated antibodies in staining buffer and then individually flow sorted into 96-well full-skirted plates (Eppendorf) containing 10µL of a 2% Dithiothreitol (DTT, 2M Sigma-Aldrich), RTL lysis buffer (Qiagen) solution. Cell lysates were sealed, mixed and spun down before storing at -80 ºC. Paired-end multiplexed sequencing libraries were then prepared following the Smart-seq2 (SS2) protocol.

Synovial tissue preparation for single-cell sequencing

	Cryopreserved knee synovial tissue biopsies were thawed then digested in digestion buffer cocktail (50ug/ml Liberase TM, Sigma and 40ug/ml DNaseI, Roche, in RPMI) in a 37°C water bath for 30 minutes with continuous stirring with a magnetic stir bar in a U-bottom polystyrene tube (12 x 75 mm2). At 15 mins during the digestion, samples were passed gently 10 times through a 14G syringe needle for additional mechanical disruption. After digestion, the cells were filtered using a 70um filter and washed prior to staining with antibodies. Following digestion, cells were counted by trypan blue to assess cell quantity and viability and loaded onto a 10x chromium controller within 5 hours of thawing.
Instrument	BD Aria II
Software	FACS Diva for aquisition, FlowJo for analysis and generation of figures.
Cell population abundance	Lymphocytes ~72% of all events; single cells ~96.6% of lymphocytes; live CD14- cells ~68% of single lymphocytes; CD3 +CD45RA- cells ~53.4% of live CD14- single lymphocytes.<br><br>Sorted fraction abundance:<br>CD4+CD8- cells ~54.6% of CD3+CD45RA-CD14- single live lymphocytes; CD8+CD4- cells ~38% of CD3+CD45RA-CD14- single live lymphocytes.<br><br>Post-sort population purity measured by re-acquiring post-sorted fractions using flow cytometry was > 99% for each sorted fraction.
Gating strategy	After using forward and side scatter area to gate on potential lymphocytes among all events, single cells gated using forward scatter area and height were further gated to only include live CD14- cells. This population was then gated to include only CD3+CD45RA- memory T cells, which was further gated to provide sorted fractions of CD4+CD8- or CD8+CD4- memory T cells.<br><br>Live CD14-, CD3+CD45RA-, CD4+CD8- and CD8+CD4- gating boundaries were drawn based on clear population separation after acquiring a small quantity of sample. Single cells were gated conservatively to exclude cells with high forward scatter area relative to height, and lymphocytes were broadly gated from all events to capture potentially larger lymphoblast cells.

☒ Tick this box to confirm that a figure exemplifying the gating strategy is provided in the Supplementary Information.
