# Supplementary material for: Single-cell sequencing reveals clonal expansions of pro-inflammatory synovial CD8 T cells expressing tissue-homing receptors in psoriatic arthritis
Source: Nat Commun. 2020 Sep 21;11:4767. doi: 10.1038/s41467-020-18513-6 (PMC7505844; doi:10.1038/s41467-020-18513-6)
Supplement: Supplementary file 4 — Description of Additional Supplementary Files [file 41467_2020_18513_MOESM4_ESM.pdf]

Description of Additional Supplementary Files

Supplementary Data 1: Cluster gene expression of all integrated datasets

Differential gene expression calculated using the Seurat FindMarkers function which utilises a 2-sided Wilcoxon Rank Sum test, run with default arguments on the following datasets:

a: Clusters identified in 3 prime 10x integrated dataset of PBMC and SFMC from patients PSA1505, PSA1607 and PSA1801 (see Supplementary Figure 2).

b: Clusters identified in 5 prime 10x integrated dataset of synovial tissue derived CD45+ leukocytes from 2 PSA patients (see Supplementary Figure 4).

c: Clusters identified in 5 prime 10x integrated dataset of PBMC and SFMC derived CD45RA-CD4+CD8- and CD45RA-CD4-CD8+ T lymphocytes from patients PSA1505, PSA1607 and PSA1801, in addition to CD45+ synovial tissue cells expressing CD3E RNA transcripts and belonging to CD3 clusters 2, 3 and 8 from Supplementary Figure 4a. Clustering of cells are visualised in Figure 2a.

d: Clusters identified in 5 prime 10x and Smart-seq 2 integrated dataset of PBMC and SFMC derived CD45RA-CD4+CD8- and CD45RA-CD4-CD8+ T lymphocytes from patient PSA1607. For each sequencing platform, cells were subsampled to include an equal number of PBMC and SFMC derived cells (relates to Figure 3h and Supplementary figure 8)

e: Clusters identified in 5 prime 10x integrated dataset of PBMC and SFMC derived CD45RA-CD4+CD8- and CD45RA-CD4-CD8+ T lymphocytes from clones enriched in either PBMC or SFMC (see Figure 5a and 5b)

f: T cells from synovial enriched clones compared to cells from blood enriched clones for the same 5 prime 10x integrated dataset referenced in (e) above.

g: All synovial fluid T cells compared to all peripheral blood T cells from the 5 prime 10x integrated dataset referenced in (c) above (excludes synovial tissue cells).

Supplementary Data 2: 10x integrated dataset cluster distribution by sample origin, patient and cell type.

Details cell composition of clusters by location from which samples were obtained, the individual from which the sample was obtained, and the cell type (CD4+/CD8+/CD4+CD8+). Relates to Figure 2a.

Supplementary Data 3: Volcano plot differentially expressed genes

Sheets a - e contain differential gene expression data on which volcano plots are based, comparing:

a: HLA-DR low CD8 cells in SFMC with HLA-DR low CD8 cells in PBMC (see Figure 2f)

b: HLA-DR high CD8 cells in SFMC with HLA-DR high CD8 cells in PBMC (see Figure 2g)

c: ZNF683+ CD8 cells in SFMC with ZNF683+ CD8 cells in PBMC (see Figure 2h)

d: HLA-DR high CD8 cells in SFMC with all other CD8 cells from other clusters in SFMC (see Figure 4e)

e: ZNF683+ CD8 cells in SFMC with all other CD8 cells from other clusters in SFMC (see Figure 4f)

Statistics for all tables calculated using 2-sided Wilcoxon Rank Sum test on the 10x 5 prime integrated dataset of all sample types subsetted to include only PBMC and SFMC derived T cells. *P*-value adjustment performed using Bonferroni correction based on total number of genes in the dataset. Seurat FindMarkers function was used, specifying assay = "RNA", min.pct = 0.01 and logfc.threshold = 0.4 as arguments.

Supplementary Data 4: Clonality per patient in both 10x and SS2 datasets

The tables within this file list clonotypes found within blood (PB), synovial synovial fluid (SF) and synovial tissue samples (ST) for each patient, determined using either 10x or Smart-seq2 processing. Clonality was determined using the 10x platform for patients PSA1505, PSA1607, PSA1801, PSA-ST1, PSA-ST2; and using Smart-seq2 for patients PSA1718, PSA1719, PSA1728, and PSA1607. Patient PSA1607 was assessed using both 10x and SS2 platforms. Clonotypes are defined by all beta and alpha chain CDR3 nucleotide sequences present within a 10x partition or Smart-seq2 well.

Headers (sheets b - i)	Description
Clonotype identifier	A clonotype identifier created by concatenating alpha and beta chain CDR3 nucleotide sequences.
fisher_p_value	*P*-value based on 2-sided Fisher's exact test
fisher_adjp	Adjusted *p*-value based on 2-sided Fisher's exact test with Benjamini & Hochberg correction

fisher_sig	Whether the proportion of this clonotype was significantly increased in blood versus synovial fluid or visa versa for a patient (using a particular platform) based on fisher_adjp value being < 0.05
Higher proportion in	Indicates whether the clone is enriched in synovial fluid or peripheral blood.
Peripheral blood frequency	Number of times this clonotype occurred in blood for this patient
Peripheral blood proportion (%)	Number of times this clonotype occurred in blood for this patient / total frequency of all clonotypes within blood for this patient
Synovial fluid frequency	Number of times this clonotype occurred in synovial fluid for this patient
Synovial fluid proportion (%)	Number of times this clonotype occurred in synovial fluid for this patient / total frequency of all clonotypes within synovial fluid for this patient
CD4 / CD8 clone type	The clone cell type assigned to this clonotype based on absolute gene expression.
beta_chain_count	The number of beta chains detected for this clonotype
beta_cdr3s_nt	The beta chain CDR3 nucleotide sequences detected for this clonotype, separated by a ";"
beta_cdr3s_aa	The beta chain CDR3 amino acid sequences detected for this clonotype, separated by a ";"
beta_vgenes	The beta chain variable genes detected for this clonotype, separated by a ";"
beta_jgenes	The beta chain joining genes detected for this clonotype, separated by a ";"
beta_dgenes	The beta chain diversity genes detected for this clonotype, separated by a ";"
beta_cgenes	The beta chain constant genes detected for this clonotype, separated by a ";"
alpha_chain_count	The number of alpha chains detected for this clonotype
alpha_cdr3s_nt	The alpha chain CDR3 nucleotide sequences detected for this clonotype, separated by a ";"
alpha_cdr3s_aa	The alpha chain CDR3 amino acid sequences detected for this clonotype, separated by a ";"
alpha_vgenes	The alpha chain variable genes detected for this clonotype, separated by a ";"
alpha_jgenes	The alpha chain joining genes detected for this clonotype, separated by a ";"
alpha_cgenes	The alpha chain constant genes detected for this clonotype, separated by a ";"

SFMC 1505 1607 1801	The convergence group, if any, this clonotype was assigned to when running the GLIPH algorithm on all CD4 and CD8 T-cells from synovial fluid for all patients processed using the 10x platform.
SFMC 1505 1607 1801 1718 1719 1728	The convergence group, if any, this clonotype was assigned to when running the GLIPH algorithm on all CD4 and CD8 T-cells from synovial fluid for all patients processed using the 10x platform, in addition to 3 independent patients processed using Smart-seq 2.

Supplementary Data 5: Cell counts and metadata of expanded peripheral blood, synovial fluid and synovial tissue clones.

a: Summary tables of all expanded CD4 and CD8 clones in both synovial fluid and blood for 3 PsA patients PSA1505, PSA1607 and PSA1801, at different stages of subsampling and filtering.

b-e: Metadata, including assigned cluster, of all PBMC and SFMC derived T cells (3 PsA patients) from the 10x Seurat integrated and subsampled dataset (Figure 2a) which formed part of a clonotype enriched in either blood or synovial fluid (Relates to Figure 5a-c).

f: Metadata, including assigned cluster, of synovial tissue T cells from the 10x Seurat integrated subsampled dataset (Figure 2a) which formed part of the largest clonotype identified in synovial tissue for patient PSA-ST2

Supplementary Data 6: GLIPH input, output and cell counts

a: The number of synovial cells represented by 10x TCR sequencing data (from 3 PsA patients) passed to GLIPH input.

b: Unique CDR3 amino acid beta chains in (a) above.

c: Input table representing all TCR beta chain CDR3 sequences discovered in the 10x synovial fluid dataset (from 3 PsA patients), passed as an argument to the GLIPH convergence group discovery algorithm (gliph-group-discovery.pl). Clonotype column denotes within patient clonotype identifier. If multiple beta chains were discovered within the same 10x partition, a "v[number]" suffix has been appended to the clonotype identifier.

d: Formatted output of GLIPH scoring algorithm (gliph-group-scoring.pl) run on results of the convergence group discovery algorithm which took (c) as input.

e: The number of cells represented by the SS2 subset (from 3 additional PsA patients) passed to second round of GLIPH input.

f: Unique CDR3 amino acid beta chains in (e) above.

g: HLA typing of patients PSA1505, PSA1607, PSA1801, PSA1718, PSA1719 and PSA1728

h: GLIPH input table, same as (c) above but additionally incorporating all SS2 identified CDR3 beta chains from patients PSA1718, PSA1719 and PSA1728.

i: Formatted output of GLIPH scoring algorithm (gliph-group-scoring.pl) run on results of the convergence group discovery algorithm which took (h) above in addition to HLA typing information obtained from patients (g) above as input.

j: HLA association with convergence groups identified by GLIPH in (i) above.

k: Comparison of CDR3 beta chain sequences from CRG-1 (derived from 10x and SS2 datasets of 6 patients) in (i) above with the CRG-CASSYSGNTEAFF precursor convergence group (derived from 10x dataset of 3 patients) in (d) above. Green highlighting indicates matching sequences in the 2 convergence groups.

Supplementary Data 7 - Cluster distribution of synovially expanded clones and CRG-1

Odds ratios and significance determined by 2-sided Fisher's exact test with Bonferroni correction (R stats package) was calculated as follows:

a: Within T cells from synovial fluid, T cells from clones enriched/unenriched in synovial fluid being part of each cluster.

b: Within CD8+ T cells from synovial fluid, CD8 T cells from CRG-1/not from CRG-1 being part of each cluster.
